# High-Efficiency Transduction of Primary Human Hematopoietic Stem/Progenitor Cells by AAV6 Vectors: Strategies for Overcoming Donor-Variation and Implications in Genome Editing

**DOI:** 10.1038/srep35495

**Published:** 2016-10-19

**Authors:** Chen Ling, Kanit Bhukhai, Zifei Yin, Mengqun Tan, Mervin C. Yoder, Philippe Leboulch, Emmanuel Payen, Arun Srivastava

**Affiliations:** 1 Division of Cellular and Molecular Therapy, Department of Pediatrics, University of Florida College of Medicine, Gainesville, FL, USA; 2 Powell Gene Therapy Center; University of Florida College of Medicine, Gainesville, FL, USA; 3 CEA/Paris Sud University (UMR-E 007), Institute of Emerging Diseases and Innovative Therapies (iMETI), Fontenay-aux-Roses, France; 4 Department of Traditional Chinese Medicine, Second Military Medical University, Shanghai, China; 5 Department of Physiology, Xiang-Ya School of Medicine, Central South University, Changsha, China; 6 Herman B Well Center for Pediatrics Research, Indiana University School of Medicine, Indianapolis, IN, USA; 7 Department of Molecular Genetics & Microbiology; University of Florida College of Medicine, Gainesville, FL, USA

## Abstract

We have reported that of the 10 commonly used AAV serotype vectors, AAV6 is the most efficient in transducing primary human hematopoietic stem/progenitor cells (HSPCs). However, the transduction efficiency of the wild-type (WT) AAV6 vector varies greatly in HSPCs from different donors. Here we report two distinct strategies to further increase the transduction efficiency in HSPCs from donors that are transduced less efficiently with the WT AAV6 vectors. The first strategy involved modifications of the viral capsid proteins where specific surface-exposed tyrosine (Y) and threonine (T) residues were mutagenized to generate a triple-mutant (Y705 + Y731F + T492V) AAV6 vector. The second strategy involved the use of *ex vivo* transduction at high cell density. The combined use of these strategies resulted in transduction efficiency exceeding ~90% in HSPCs at significantly reduced vector doses. Our studies have significant implications in the optimal use of capsid-optimized AAV6 vectors in genome editing in HSPCs.

Genetically-modified autologous hematopoietic stem/progenitor cells (HSPCs) transplantation is the most promising therapeutic strategy to treat inherited genetic diseases, such as β-globin disorders^1^, leukodystrophies^2^,^3^, severe combined immunodeficiency^4^, Wiskott-Aldrich syndrome^5^, as well as acquired diseases such as AIDS^6^. Recent remarkable progress in genome editing tools include zinc-finger nucleases (ZFNs), transcription activator like effector nucleases (TALENs), and the RNA-guided clustered regulatory interspaced short palindromic repeat (CRISPR)/Cas9 endonucleases. These targeted nucleases further expand the application of *ex vivo* editing of therapeutic genes into patient HSPCs^7^. Most of these methods require the delivery of extracellular DNA into HSPCs, the efficiency of which is frequently sub-optimal. In the present study, we describe a novel, adeno-associated virus (AAV) vector-based DNA delivery strategy to overcome the current drawbacks that limit the clinical use of HSPCs gene editing methods.

The current drawbacks of HSPCs gene editing using viral methods such as chimeric adenoviral vectors, retroviral vectors, or integrase-defective lentiviral vectors (IDLV) include inefficient gene delivery, cytotoxicity, or DNA insertion. For example in a phase I/II clinical trial, a self-inactivating lentiviral vector achieved genetic modification in only 9–14% of blood cells after transplant^2^. Similarly, in laboratory, lentiviral transduction can produce no more than 22% transgene expression even in the presence of the best chemical helper^8^. Although it has now become feasible to achieve significantly higher transduction efficacy in HSPCs because of lentiviral vector optimization^9^ and good manufacturing process^10^, the high rate of stable transduction is also concomitant with a high vector genome copy number per cell, which, raises safety concerns because lentiviral genome, unlike the AAV genome, is associated with high risk of insertional mutagenesis. Non-integrative lentiviral vectors are safer but still limited by lower level of gene expression than other systems^11^. Cytotoxicity concerns also remains on transduced HSPCs. For example, the combination of the adenoviral vector and a potential enhancer, protein kinase C, resulted in >25% gene disruption, however, it is less tolerated in HSPCs than in other cell types^12^. Theoretically, the presence of any DNA material may have a chance to insert into the host genome. Insertion is a potential problem as extended expression of the nuclease may increase off-targeting. In addition, the nature of viral-based vectors, such as lentiviral, retroviral, and adenoviral vectors, enhance the random insertional activities that may lead to altered gene expression. For example, in a recent clinical trial using retroviral vectors, >140,000 unambiguous integration sites and a polyclonal pattern of hematopoiesis were revealed in all patients, which led to acute leukemia in 7 of 10 patients^13^. However, editing for therapeutic purposes in HSPCs will be done with non-integrative vectors. Although the probability of IDLV vectors to integrate in a cell is much lower than for normal integrative lentiviral vectors, and AAV vectors have been shown to undergo integration, albeit at a much lower frequency, the rates of integration for IDLV and AAV vectors remain to be directly compared.

Although vectors based on AAV2 serotype have been used for transduction of primary human HSPCs, the efficiency has been reported to be low^14^,^15^,^16^. Based on our initial studies, in which we mutagenized the surface-exposed tyrosine (Y) residues on AAV2 capsids, and observed a significant increase in their transduction efficiency^17^,^18^, the transduction efficiency of primary human HSPCs could be further increased with the capsid-modified AAV2 vectors^19^. As additional AAV serotypes became available, we systematically evaluated the transduction efficiency of the 10 most commonly used AAV serotype vectors in primary human HSPCs, and observed that the AAV6 serotype vectors were the most efficient^20^, and that the transduction efficiency of capsid-modified AAV6 vectors could be increased significantly^21^.

In our present studies, we describe two additive strategies to augment the transduction efficiency of AAV6 serotype vectors in primary human CD34^+^ cells, as well as propose a hypothetical model to help explain the underlying molecular mechanism of the observed increase in transduction. Our studies have not only contributed to a broader understanding of the AAV-host cell interactions, but have also led to the development of novel AAV6 serotype vectors for their optimal use in genome editing in primary human hematopoietic stem/progenitor cells.

## Results

**Figure 1:**
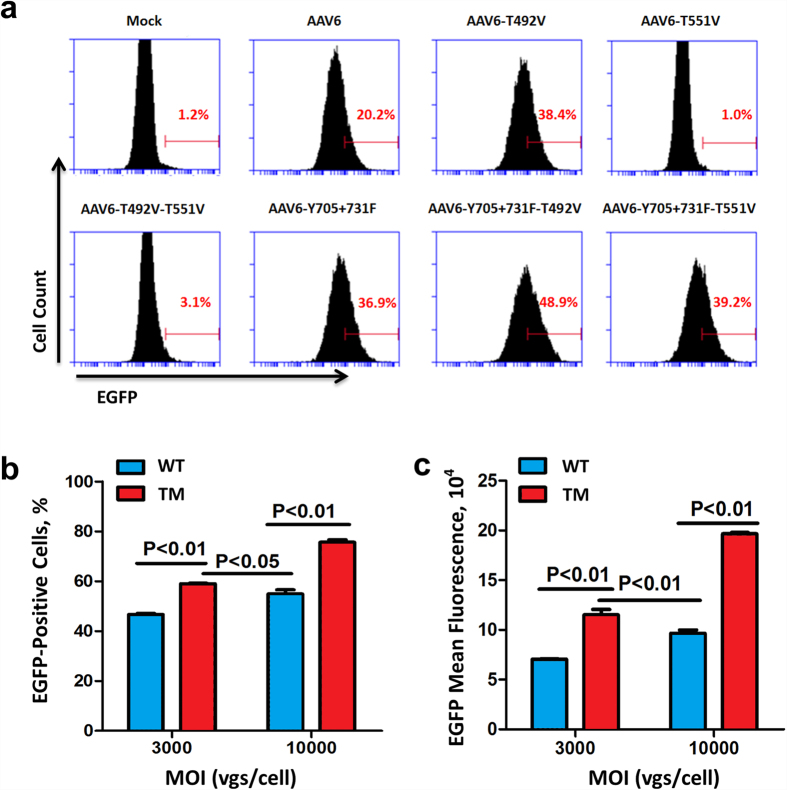
Transduction efficiency of various capsid-modified AAV6 vectors in human hematopoietic cells. **(a)** Human erythroleukemia cells, K562, were either mock-transduced, or transduced with the wild-type (WT) or various indicated capsid-modified scAAV6-CBAp-EGFP vectors at an MOI of 3,000 vgs/cell. Transgene expression was analyzed by flow cytometry 48 hrs post-transduction. **(b)** Primary human bone marrow-derived CD34^+^ cells from a single individual donor were transduced in triplicate with the WT or the Y705 + 731F + T492V triple-mutant (TM) scAAV6-CBAp-EGFP vectors at MOIs of 3,000 or 10,000 vgs/cell. Transgene expression was analyzed by flow cytometry 48 hrs post-transduction. **(c)** Mean fluorescence intensity of transgene expression. Error bars represent standard deviations (SD).

Our previous efforts have identified recombinant AAV serotype 6 (rAAV6) with high tropism for human HSPCs^20^,^21^. We have also reported that the transduction efficiency ranged between ~0–50% (n = 12 donors) for AAV2, and ~6–87% (n = 11 donors) for AAV6 in HSPCs, respectively^15^,^20^. Although we hypothesized that such a wide range of donor variation is due to different levels of expression of the putative receptors and/or co-receptors on these cells, we were able to achieve significant increase in the transduction efficiency of both AAV2 and AAV6 vectors using site-directed mutagenesis of surface-exposed tyrosine (Y) residues. We therefore wished to examine whether the transduction efficiency of AAV6 vectors could be further improved by additional capsid modifications involving mutagenesis of surface-exposed serine (S) and threonine (T) residues, and various permutations and combinations thereof^22^. To this end, a number of such mutant capsids were generated, and self-complementary AAV6 (scAAV6) vectors containing a chicken beta actin promoter/CMV enhancer driving an enhanced green fluorescence protein gene (CBAp-EGFP) were produced as previously reported^23^. Using K562 cells, frequently used as a model for hematopoietic cell transduction studies, indicated that among all AAV6 capsid variants tested, a triple-mutant (TM) AAV6-Y705 + 731F + T492V (TM-AAV6) vector was identified as the most efficient (Fig. 1a). The transduction efficiency of the TM-AAV6 vectors was also consistently significantly higher than its wild-type (WT) counterpart not only in K562 cells, but also in primary human bone marrow-derived HSPCs obtained from a normal volunteer donor (Fig. 1b,c). Similar results were obtained with HSPCs obtained from one additional donor (Supplementary Table 1, Donor 2). No variability in expression levels under different transduction conditions was observed using flow cytometry or fluorescence microscopy.

**Figure 2:**
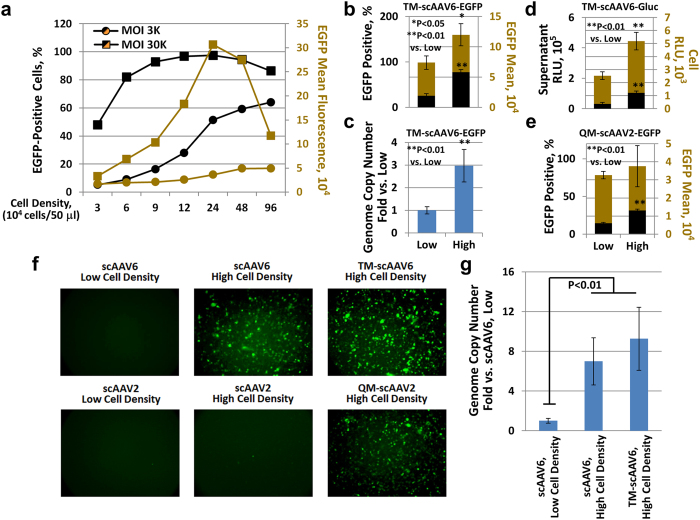
Transduction efficiency of AAV vectors in human hematopoietic cells at various cell densities. **(a)** K562 cells were transduced at various indicated cell densities at MOIs of 3,000 or 30,000 vgs/ml with WT scAAV6-CBAp-EGFP vectors. Transgene expression was analyzed by FACS 48 hrs post-transduction. **(b)** K562 cells were also transduced at low (1 × 10^6^/mL) or high (1 × 10^7^/mL) cell densities with 3,000 vgs/cell of TM scAAV6-CBAp-EGFP vectors, and transgene expression and mean fluorescence intensity were determined as described above. **(c)** The vector genome copy numbers/cell were determined 2 hrs post-vector transduction by qPCR and data were normalized to β-actin DNA copy number. **(d)** K562 cells were transduced at low or high cell densities with TM-scAAV6-CBAp-Gluc vectors, and luciferase expression was determined in the culture supernatants. **(e)** K562 cells were transduced at low or high cell densities with QM-scAAV2-CBAp-EGFP vectors, and transgene expression and mean fluorescence intensity were determined as described above. **(f)** Primary human bone marrow-derived CD34^+^ cells were transduced at low or high densities with indicated AAV6 or AAV2 vectors, EGFP-expressing cells were visualized under a fluorescence microscope 48 hrs post-transduction. **(g)** The vector genome copy numbers/cell were determined as described above.

During the course of these studies, we also observed that the transduction efficiency of the WT AAV6 vectors could be significantly increased if transductions were carried at high cell density, both at low and high multiplicities of infection (MOIs), in contrast to previously published studies, in which transductions were carried out at cell densities ranging from 7.0 × 10^5^–1.1 × 10^6 ^cells/ml^14^,^15^,^16^,^19^,^20^,^21^,^24^. The results, as analyzed by flow cytometry 48 hrs post-transduction, indicated that, compared to the conventionally used cell densities, increased cell density, up to 1.0 × 10^7^ cells/ml, dramatically enhanced the scAAV6-mediated transgene expression, in both the EGFP-positivity and EGFP mean fluorescence intensity (Fig. 2a), presumably due to the increased probability of more efficient vector attachment to the cell receptor and/or co-receptor. Next, K562 cells were transduced with the optimized TM-scAAV6-CBAp-EGFP vectors either at low-density (1 × 10^6 ^cells/ml) or high-density (1 × 10^7 ^cells/ml). Whereas only ~25% of K562 cells were transduced at low-density, the transduction efficiency at high-density increased up to 77%, and the EGFP mean value increased to 160% (Fig. 2b,c). The enhancement of transgene expression also correlated with a significantly increased intra-cellular viral genome copy number (Fig. 2c), as determined by qPCR of total DNA isolated 2 hrs post-transduction^25^. Similar results were obtained with the TM-scAAV6 vectors expressing the Gaussia luciferase (Gluc) transgene (Fig. 2d), as well as when the optimized AAV2-CBAp-EGFP vectors containing the quadruple mutation (Y444 + 500 + 730F + T491V; QM-scAAV2) vectors, which we have previously identified to be the most efficient vector, albeit in human cell lines *in vitro*, and in murine hepatocytes *in vivo*^26^, were used (Fig. 2e). Similar results were also obtained when these serotype vectors were used to transduce HSPCs from a donor, which are transduced extremely poorly under conventional conditions (Fig. 2f). The enhanced transgene expression mediated by TM-scAAV6 vectors observed under high cell density conditions also correlated well with increased internalized vector genome copy number/cell (Fig. 2g) as well as with ssAAV6 vectors (Supplementary Fig. 1a).

To further corroborate our hypothesis, K562 cells were transduced and later concentrated or diluted. That the initial cell-cell contact was critical in achieving high-efficiency transduction, was further corroborated by experiments in which cells were transduced at low-density, and subsequently pooled together and centrifuged to reach high-density, and conversely, cells were transduced at high-density, and soon after transduction, were diluted to low-density (Supplementary Fig. 2a). The elevated transduction was observed only under the latter condition (Supplementary Fig. 2b). The increased transduction efficiency at high-density also correlated well with the vector genomes entering the cells, as determined by qPCR analyses (Supplementary Fig. 1a). In the second set of experiments, a fixed number of K562 cells were infected with viral vectors in various volumes for 2 hrs and subsequently diluted in the same volume of 2 ml (Supplementary Fig. 3a). Once again, the increased transduction efficiency was observed only under the condition of high cell density (Supplementary Fig. 3b,c), accompanied with a significantly increased intra-cellular viral genome copy numbers (Supplementary Fig. 3d).

We next extended these studies to include two additional human hematopoietic cell lines, M07e and Raji, which express low to extremely low levels of HSPG, the primary receptor for AAV2, and consequently, are transduced extremely poorly by AAV2 vectors^27^. As can be seen in Supplementary Fig. 4a,b, under the condition of high cell density, significantly enhanced transduction of M07e cells, but not Raji cells, was observed, since M07e cells do express high levels of AAV2 co-receptor, fibroblast growth factor receptor 1 (Supplementary Fig. 4c), but not Raji cells, which express undetectable levels of both HSPG and FGFR1^27^. To address the possibility whether alternative receptors/co-receptors were being used under the condition of high cell density, K562 cells were transduced with scAAV2 vectors in the absence or the presence of heparin, which is known to compete for AAV2 cellular entry. As can be seen in Supplementary Fig. 4d,e, heparin at 5 μg/ml significantly reduced the transduction efficiency of scAAV2 vectors under the condition of high cell density for each of the cell types tested. These results strongly suggest that the putative receptors/co-receptors for viral entry remain unaltered under the condition of high cell density.

**Figure 3:**
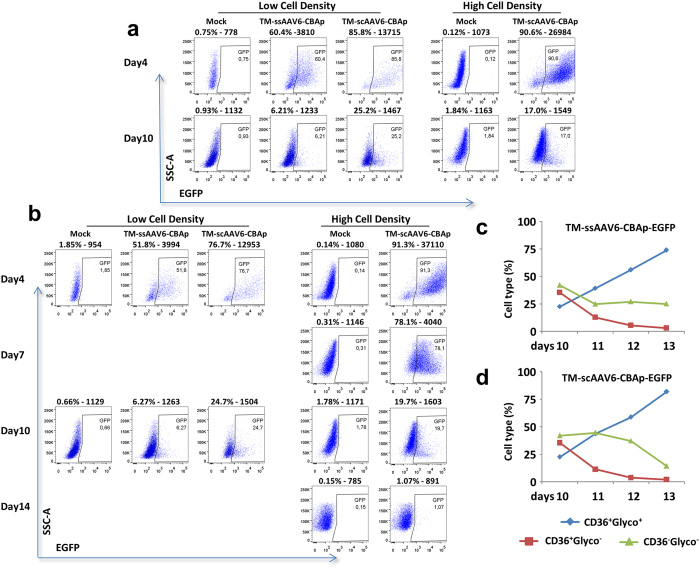
Transduction efficiency of TM-ssAAV6 and TM-scAAV6 vectors in primary human CD34^+^ cells. **(a)** Primary human cord blood-derived CD34^+^ cells were either mock-transduced, or transduced at day 0 at low (0.5 × 10^6 ^cells/ml,) or high (1 × 10^7^ cells/ml) cell density with 20,000 vgs/cell of the indicated AAV6 vectors in serum free XVIVO20 medium. Two hrs later, cells were diluted to 5 × 10^5 ^cells/mL and switched to the expansion medium (IMDM + FBS + SCF + IL3 + Epo+ Dexamethasone + β-estradiol + β-mercapthoethanol). EGFP expression was determined by flow cytometry at day 4 and day 10 post-transduction. **(b)** Following mock-transduction, or transduction of CD34^+^  cells as described above, cells were switched to the expansion medium for 10 days, and cultured in an erythroid differentiation medium (IMDM + BSA + Insulin + Transferrin + Epo) for an additional four days. EGFP expression was determined by flow cytometry. **(c**,**d)** Vector-transduced CD34^+^ cells cultured in the differentiation medium were stained with hCD36-PE and hGlycophorin A^−^FITC and analyzed by flow cytometry for the following: non-erythroid (CD36^−^/glycoA^−^), and erythroid cells (CD36^+^/GlycoA^+^) from day 10 to day 14.

We further evaluated the efficacy of AAV vector-mediated transduction of primary HSPCs derived from bone marrow (BM) as well as from umbilical cord blood (CB). BM-derived CD34^+^ cells from individual donors (or a mixture from 10 donors) were purchased from a commercial source (AllCells, LLC, Alameda, CA, USA), and were used to transduce with different scAAV-CBAp-EGFP vectors at an MOI of 10,000 vgs/cell without fetal bovine serum (FBS). Transgene expression was evaluated by flow cytometry 48 hrs post-transduction. As shown in Supplementary Table 1, consistent with our previously published studies, whereas scAAV6 vectors transduced human HSPCs more efficiently than scAAV2 vectors, capsid modification on both vectors further enhanced their transduction efficiency. The transgene expression at high cell density was consistently higher than that at low cell density. The increased transduction efficiency in human HSPCs at high cell density also correlated with a significantly increased intra-cellular viral genome copy number 2 hrs post-viral transduction (Supplementary Fig. 1b). However, the extent of transgene expression declined over time, and in none of the cell populations tested, the viral genome copy number was above the detection limit of qPCR 14 days post-transduction (data not shown). Similar results were obtained with CB-derived CD34^+^ cells, except that the overall transduction efficiency was consistently higher than that in BM-derived CD34^+^ cells. In the first set of experiments, CD34^+^ cells were transduced at low and high cell densities with TM-scAAV6-CBAp-EGFP and TM-ssAAV6-CBAp-EGFP vectors at an MOI of 20,000 vgs/cell without FBS, followed by switch to FBS-containing expansion medium and cultured for 10 days, and transgene expression was evaluated by FACS at 4 and 10 days post-transduction (Fig. 3a). It is evident that at day 4, scAAV6 vectors were more efficient than ssAAV6 vectors, but at high cell density, there was only a modest enhancement in EGFP-positivity. Again, the transgene expression diminished over time. In the second set of experiments, CB-derived CD34^+^ cells were transduced at low and high cell densities with TM-scAAV6-CBAp-EGFP vectors as described above, except that at day 10, cells were switched to an erythroid differentiation medium and cultured for 4 additional days. EGFP expression was determined by flow cytometry at day 4, day 7, day 10, and day 14 (Fig. 3b). As can be seen, once again, at day 4, scAAV6 vectors were more efficient than ssAAV6 vectors. At high cell density, there was a modest enhancement in EGFP-positivity, but transgene expression diminished over time, and eventually resulted in less than 1% EGFP-positive cells 14 days post-transduction, which is within the variation of mock-transduced cells (0.14–1.84%), corroborating again the loss of vector genome, as determined by qPCR assays described above, and hence, lack of stable integration in HSPCs. Staining with hCD36-PE and hGlycophorin A-FITC showed the typical induction of erythroid cell differentiation, which was unaltered following vector transduction (Fig. 3c,d). At the end of the culture period, most cells were GlycoA^+^, indicating that none of erythroid-differentiated cells expressed the transgene.

## Discussion

Although AAV vectors have been successfully used in transducing a wide variety of cells and tissues, primary hematopoietic stem/progenitor cells (HSPCs) have proven to be particularly difficult to transduce despite sustained efforts by us and others spanning nearly three decades. However, most of those studies were carried out with AAV2 serotype vectors. With the subsequent availability of a number of additional serotypes, we were able to identify AAV6 as the most efficient in transducing HSPCs^20^,^21^. Despite this advance, we also observed a significant donor-variation ranging between 6–87%. Although this could be due to differences in various steps in the life cycle of AAV6 vectors in HSPCs from different donors, we chose to focus on the initial virus-host cell interaction, primarily involving the putative cellular receptors and co-receptors for AAV6. Although epidermal growth factor receptor (EGFR) has been identified to be the cellular receptor for AAV6^28^, those studies were not performed with hematopoietic cells. In addition, we have previously reported that K562 cells, which are known to lack expression of EGFR, are efficiently transduced by AAV6 vectors, and that pre-treatment of primary human CD34^+^ cells with EGF has no effect on the transduction efficacy of AAV6 vectors^21^. Thus, the precise identity of the receptor and co-receptor for AAV6 in HSPCs remains elusive. Based on our observation of significantly increased transduction under the conditions of high cell density, we hypothesized that a close cell-cell contact facilitates more efficient vector binding and entry into cells grown in suspension culture, presumably because of the close proximity of the receptor and the co-receptor on neighboring cells, and AAV6 vector bound to the receptor on one cell utilizes the co-receptor on the neighboring cell to gain entry in the latter, and vice versa, thus leading to increased transduction. Indeed, using AAV2 vectors, for which the cellular receptors and co-receptors have been well characterized, we were able to corroborate our hypothesis to a certain extent.

Clearly, additional studies are warranted, but we believe that our data have revealed a novel mechanism, which AAV vectors exploit to gain entry into target cells. For example, adherent cells such as HEK293, are readily transduced by AAV2 vectors because these cells abundantly express the cell surface receptor, heparan sulfate proteoglycan (HSPG), and one of the co-receptors, human fibroblast growth factor receptor 1 (FGFR1) for AAV2. However, K562 cells are not transduced as efficiently, although these cells, grown in suspension, express both HSPG and FGFR1 only at modestly lower levels. We reasoned that the lack of proximity of HSPG and FGFR1 on K562 cells might account for the suboptimal transduction of these cells, and hypothesized that if the transduction was performed at high cell density, presumably allowing for HSPG on one cell to come in close proximity to FGFR1 on the neighboring cell, then AAV2 bound to HSPG on one cell could utilize FGFR1 on the neighboring cell to gain entry in the latter, and vice versa, thus leading to increased transduction which was observed experimentally.

We also believe that our studies described here have practical utility. For example, recent development of targeted nucleases provides a means to achieve precise genome editing in stem cells, including human HSPCs. However, all these strategies require the delivery of DNA materials. Although the expression of targeted nucleases could be achieved by mRNA delivery, a homologous donor DNA template must also be introduced. An exception is the CRISPR/Cas9 system that utilizes a short guide RNA and RNA-guided nuclease^29^. Nevertheless, the majority of research still uses DNA delivery systems, such as plasmids and viral vectors to express the short guide RNA, due to the low efficiency of RNA delivery in human HSPCs^30^. Thus, the off-target activities as well as random insertion of the delivered DNA constitute significant safety hurdles to overcome^31^. While our current studies were in progress, three independent groups not only corroborated our previously published studies^20^,^21^, but also reported successful transduction of primary human CD34^+^ cells using the WT AAV6 vectors for genome editing^32^,^33^,^34^, except that MOIs ranging from 100,000–200,000 vgs/cell were used to achieve ~40–55% transduction efficiency. Using capsid-optimized AAV6 vectors and transduction conditions described here, at an MOI of 20,000 vgs/cell, transduction efficiency exceeding 90% can now routinely be achieved, which is significantly higher than any known vector system currently in use. Thus, our studies have significant implications in highly efficient gene therapy and genome editing with capsid-optimized AAV6 vectors involving primary human HSPCs.

## Methods

### Cells and reagents

Human embryonic kidney cell line, HEK293, and human erythroleukemia cell line, K562, were purchased from the American Type Culture Collection (Manassas, VA). The human lymphoblastoid cell line, Raji, and the human megakaryocytic leukemia cell line, M07e, were obtained from Drs Z.A. Brahmi and H.E. Broxmeyer (Indiana University School of Medicine, Indianapolis, IN), respectively. Monolayer cultures of HEK293 were maintained in Dulbecco’s-modified Eagle’s medium (DMEM), and suspension cultures of K562, M07e, and Raji were maintained in Iscove’s-modified Dulbecco’s medium (IMDM) supplemented with 10% fetal bovine serum (FBS) and 1% antibiotics (Lonza, Basel, Switzerland). Primary human bone marrow-derived CD34^+^ cells were purchased from AllCells (Alameda, CA). Human cord blood cells were collected by the Assistance Publique – Hôpitaux de Paris (AP-HP), according to French bioethics law 2011-814. The research project for cell usage was approved by the scientific council of Fondation Générale de Santé (FGDS) and AP-HP. Written informed consents were obtained from mothers of the newborn.

### AAV vector production

The ssAAV and scAAV plasmids containing the CBAp-EGFP transgene expression cassette have been described previously^35^. All AAV vectors were produced using the polyethelenimine-mediated triple-plasmid transfection method^36^. Briefly, HEK293 cells (Agilent Technologies) were harvested 72 hrs post-transfection, and lysed by 3 rounds of freeze-thaw, and digested with Benzonase (Invitrogen, Grand Island, NY). Cell debris was removed by centrifugation. AAV vectors were purified by iodixanol (Sigma, St. Louis, MO) gradient ultracentrifugation, followed by ion exchange chromatography using HiTrap SP/Q HP columns (GE Healthcare, Piscataway, NJ), washed with phosphate-buffered saline (PBS) and concentrated by centrifugation using centrifugal spin concentrators with 150 K molecular weight cutoff. Titers were determined by Quantitative real-time PCR assays as previously described^37^.

### AAV transduction

The culture conditions for K562, Raji, and M07e cell have been described previously^27^. AAV transductions were performed in FBS-free medium for 2 hrs. Cells were then switched to FBS-containing medium for growth. Primary human HSPCs were purchased from AllCells Technologies (Emeryville, CA). Cord blood CD34^+^ cells were thawed and immediately transduced with AAV vectors at 20,000 vgs/cell in serum free XVIVO20 medium. Two hrs post-transduction, cells were switched to the expansion medium (IMDM, FBS, SCF, IL3, Epo, Dexamethasone, β-estradiol, β-mercapthoethanol) and grown at 5 × 10^5^ cells/mL. At day 10, cells were switched to the erythroid differentiation medium (IMDM, BSA, Insulin, Transferrin, Epo). All transgene expressions were determined either by fluorescence microscopy or by flow cytometry, as described previously^20^,^21^.

### Erythroid expansion of CD34^+^ cells

Ten days after culturing in the expansion medium, cells were stained with hCD36-PE and hGlycophorin A-FITC showing the typical profile of expanded cells in this amplification medium: non-erythroid (CD36^−^/glycoA^−^), and erythroid cells (immature: CD36^+^/GlycoA^−^; early mature: CD36^high^/GlycoA^med^ and late mature: CD36^med^/glycoA^high^). Stained cells were subjected to flow cytometry assays.

### Viral genome copy number determination

Total cellular DNA was isolated using the PureLink^®^ Genomic DNA Kits (ThermoFisher Scientific, MA) in accordance with the manufacturer’s protocol. DNA concentration was determined by Nanodrop (ThermoFisher Scientific, MA). One hundred ng of DNA from each sample was used as the template material for quantitative real-time PCR. The EGFP vector genome copy numbers were determined and normalized by the results of human β-actin gene in each sample.

### Heparin competition assays

Cells were transduced with scAAV2-CBAp-EGFP vectors at an MOI of 5,000 vgs/cell. Vectors were premixed with vehicle control or 5 μg/ml heparin sodium salt (Sigma, St. Louis, MO). Cells were switched to heparin-free complete DMEM 2 hrs post-transduction and then were analyzed for EGFP levels by flow cytometry 72 hrs post-transduction.

## Additional Information

**How to cite this article**: Ling, C. *et al*. High-Efficiency Transduction of Primary Human Hematopoietic Stem/Progenitor Cells by AAV6 Vectors: Strategies for Overcoming Donor-Variation and Implications in Genome Editing. *Sci. Rep.* **6**, 35495; doi: 10.1038/srep35495 (2016).

## Supplementary Material

Supplementary Information

## References

[b1] Cavazzana-Calvo M. . Transfusion independence and HMGA2 activation after gene therapy of human beta-thalassaemia. Nature 467, 318–322 (2010). 20844535 10.1038/nature09328 PMC3355472

[b2] Cartier N. . Hematopoietic stem cell gene therapy with a lentiviral vector in X-linked adrenoleukodystrophy. Science 326, 818–823 (2009). 19892975 10.1126/science.1171242

[b3] Biffi A. . Lentiviral hematopoietic stem cell gene therapy benefits metachromatic leukodystrophy. Science 341, 1233158 (2013). 23845948 10.1126/science.1233158

[b4] Hacein-Bey-Abina S. . Efficacy of gene therapy for X-linked severe combined immunodeficiency. N Engl J Med 363, 355–364 (2010). 20660403 10.1056/NEJMoa1000164 PMC2957288

[b5] Aiuti A. . Lentiviral hematopoietic stem cell gene therapy in patients with Wiskott-Aldrich syndrome. Science 341, 1233151 (2013). 23845947 10.1126/science.1233151 PMC4375961

[b6] DiGiusto D. L. . RNA-based gene therapy for HIV with lentiviral vector-modified CD34(+) cells in patients undergoing transplantation for AIDS-related lymphoma. Sci Transl Med 2, 36ra43 (2010). 10.1126/scitranslmed.3000931 PMC3130552 20555022

[b7] Gaj T., Gersbach C. A. & Barbas C. F. 3rd ZFN, TALEN, and CRISPR/Cas-based methods for genome engineering. Trends Biotechnol 31, 397–405 (2013). 23664777 10.1016/j.tibtech.2013.04.004 PMC3694601

[b8] Johnston J. M. . High-throughput screening identifies compounds that enhance lentiviral transduction. Gene Ther 21, 1008–1020 (2014). 25231175 10.1038/gt.2014.80

[b9] Negre O. . Preclinical evaluation of efficacy and safety of an improved lentiviral vector for the treatment of beta-thalassemia and sickle cell disease. Curr Gene Ther 15, 64–81 (2015). 25429463 10.2174/1566523214666141127095336 PMC4440358

[b10] Merten O. W. . Large-scale manufacture and characterization of a lentiviral vector produced for clinical ex vivo gene therapy application. Hum Gene Ther 22, 343–356 (2011). 21043787 10.1089/hum.2010.060

[b11] Joglekar A. V. . Integrase-defective lentiviral vectors as a delivery platform for targeted modification of adenosine deaminase locus. Mol Ther 21, 1705–1717 (2013). 23857176 10.1038/mt.2013.106 PMC3776625

[b12] Li L. . Genomic editing of the HIV-1 coreceptor CCR5 in adult hematopoietic stem and progenitor cells using zinc finger nucleases. Mol Ther 21, 1259–1269 (2013). 23587921 10.1038/mt.2013.65 PMC3677314

[b13] Braun C. J. . Gene therapy for Wiskott-Aldrich syndrome–long-term efficacy and genotoxicity. Sci Transl Med 6, 227ra233 (2014). 10.1126/scitranslmed.3007280 24622513

[b14] Zhou S. Z. . Adeno-associated virus 2-mediated high efficiency gene transfer into immature and mature subsets of hematopoietic progenitor cells in human umbilical cord blood. J Exp Med 179, 1867–1875 (1994). 7515101 10.1084/jem.179.6.1867 PMC2191525

[b15] Ponnazhagan S. . Adeno-associated virus type 2-mediated transduction in primary human bone marrow-derived CD34+ hematopoietic progenitor cells: donor variation and correlation of transgene expression with cellular differentiation. J Virol 71, 8262–8267 (1997). 9343178 10.1128/jvi.71.11.8262-8267.1997 PMC192284

[b16] Chatterjee S. . Transduction of primitive human marrow and cord blood-derived hematopoietic progenitor cells with adeno-associated virus vectors. Blood 93, 1882–1894 (1999). 10068661

[b17] Zhong L. . Next generation of adeno-associated virus 2 vectors: point mutations in tyrosines lead to high-efficiency transduction at lower doses. Proc Natl Acad Sci USA 105, 7827–7832 (2008). 18511559 10.1073/pnas.0802866105 PMC2402387

[b18] Markusic D. M. . High-efficiency transduction and correction of murine hemophilia B using AAV2 vectors devoid of multiple surface-exposed tyrosines. Mol Ther 18, 2048–2056 (2010). 20736929 10.1038/mt.2010.172 PMC2997584

[b19] Kauss M. A. . Enhanced long-term transduction and multilineage engraftment of human hematopoietic stem cells transduced with tyrosine-modified recombinant adeno-associated virus serotype 2. Hum Gene Ther 21, 1129–1136 (2010). 20486772 10.1089/hum.2010.016 PMC2936497

[b20] Song L. . Optimizing the transduction efficiency of capsid-modified AAV6 serotype vectors in primary human hematopoietic stem cells *in vitro* and in a xenograft mouse model *in vivo*. Cytotherapy 15, 986–998 (2013). 23830234 10.1016/j.jcyt.2013.04.003 PMC3711144

[b21] Song L. . High-efficiency transduction of primary human hematopoietic stem cells and erythroid lineage-restricted expression by optimized AAV6 serotype vectors *in vitro* and in a murine xenograft model *in vivo*. PLoS One 8, e58757 (2013). 23516552 10.1371/journal.pone.0058757 PMC3597592

[b22] Srivastava A. Adeno-Associated Virus: The Naturally Occurring Virus Versus the Recombinant Vector. Hum Gene Ther 27, 1–6 (2016). 26784640 10.1089/hum.2015.29017.asr PMC4809066

[b23] Wang L. N. . Pristimerin enhances recombinant adeno-associated virus vector-mediated transgene expression in human cell lines *in vitro* and murine hepatocytes *in vivo*. J Integr Med 12, 20–34 (2014). 24461592 10.1016/S2095-4964(14)60003-0

[b24] Smith L. J. . Gene transfer properties and structural modeling of human stem cell-derived AAV. Mol Ther 22, 1625–1634 (2014). 24925207 10.1038/mt.2014.107 PMC4435483

[b25] Zhang Y. H. . Cytotoxic genes from traditional Chinese medicine inhibit tumor growth both *in vitro* and *in vivo*. J Integr Med 12, 483–494 (2014). 25412666 10.1016/s2095-4964(14)60057-1

[b26] Aslanidi G. V. . Optimization of the capsid of recombinant adeno-associated virus 2 (AAV2) vectors: the final threshold? PLoS One 8, e59142 (2013). 23527116 10.1371/journal.pone.0059142 PMC3602601

[b27] Qing K. . Human fibroblast growth factor receptor 1 is a co-receptor for infection by adeno-associated virus 2. Nat Med 5, 71–77 (1999). 9883842 10.1038/4758

[b28] Weller M. L. . Epidermal growth factor receptor is a co-receptor for adeno-associated virus serotype 6. Nat Med 16, 662–664 (2010). 20473307 10.1038/nm.2145 PMC2885716

[b29] Hendel A. . Chemically modified guide RNAs enhance CRISPR-Cas genome editing in human primary cells. Nat Biotechnol (2015). 10.1038/nbt.3290 PMC4729442 26121415

[b30] Schmidt F. & Grimm D. CRISPR genome engineering and viral gene delivery: a case of mutual attraction. Biotechnol J 10, 258–272 (2015). 25663455 10.1002/biot.201400529

[b31] Gammon K. Gene therapy: editorial control. Nature 515, S11–S13 (2014). 25390136 10.1038/515S11a

[b32] Sather B. D. . Efficient modification of CCR5 in primary human hematopoietic cells using a megaTAL nuclease and AAV donor template. Sci Transl Med 7, 307ra156 (2015). 10.1126/scitranslmed.aac5530 PMC4790757 26424571

[b33] Wang J. . Homology-driven genome editing in hematopoietic stem and progenitor cells using ZFN mRNA and AAV6 donors. Nat Biotechnol 33, 1256–1263 (2015). 26551060 10.1038/nbt.3408 PMC4842001

[b34] De Ravin S. S. . Targeted gene addition in human CD34 hematopoietic cells for correction of X-linked chronic granulomatous disease. Nat Biotechnol 34, 424–431 (2016). 26950749 10.1038/nbt.3513 PMC4824656

[b35] Ma W. . A simple method to increase the transduction efficiency of single-stranded adeno-associated virus vectors *in vitro* and *in vivo*. Hum Gene Ther 22, 633–640 (2011). 21219084 10.1089/hum.2010.243 PMC3081437

[b36] Dong B. . A concept of eliminating nonhomologous recombination for scalable and safe AAV vector generation for human gene therapy. Nucleic Acids Res 41, 6609–6617 (2013). 23677609 10.1093/nar/gkt404 PMC3711426

[b37] Wang Q. . Efficient production of dual recombinant adeno-associated viral vectors for factor VIII delivery. Hum Gene Ther Methods 25, 261–268 (2014). 25093498 10.1089/hgtb.2014.093 PMC4142791

