# Supplementary material for: High-Efficiency Transduction of Primary Human Hematopoietic Stem/Progenitor Cells by AAV6 Vectors: Strategies for Overcoming Donor-Variation and Implications in Genome Editing
Source: Sci Rep. 2016 Oct 19;6:35495. doi: 10.1038/srep35495 (PMC5069717; doi:10.1038/srep35495)
Supplement: Supplementary Information [file srep35495-s1.pdf]

SUPPLEMENTARY INFORMATION

High-Efficiency Transduction of Primary Human Hematopoietic Stem/Progenitor Cells by AAV6 Vectors: Strategies for Overcoming Donor-Variation and Implications in Genome Editing

Chen Ling[1,2†], Kanit Bhukhai[3†], Zifei Yin[1,2,4†], Mengqun Tan[5], Mervin C. Yoder[6], Philippe Leboulch[3]*, Emmanuel Payen[3]*, and Arun Srivastava[1,2,7]*

[1]Division of Cellular and Molecular Therapy, Department of Pediatrics; [2]Powell Gene Therapy Center; University of Florida College of Medicine, Gainesville, FL, USA; [3]CEA/Paris Sud University (UMR-E 007), Institute of Emerging Diseases and Innovative Therapies (iMETI), Fontenay-aux-Roses, France; [4]Department of Traditional Chinese Medicine, Second Military Medical University, Shanghai, China; [5]Department of Physiology, Xiang-Ya School of Medicine, Central South University, Changsha, China; [6]Herman B Well Center for Pediatrics Research, Indiana University School of Medicine, Indianapolis, IN, USA; [7]Department of Molecular Genetics & Microbiology; University of Florida College of Medicine, Gainesville, FL, USA

[†]These authors contributed equally to this work

*Corresponding authors:

Arun Srivastava, PhD
Division of Cellular and Molecular Therapy, Departments of Pediatrics, Molecular Genetics & Microbiology, Powell Gene Therapy Center, Cancer and Genetics Research Complex, 2033 Mowry Road, Room 492-A, University of Florida College of Medicine, Gainesville, FL 32611-3633; USA; E-mail: aruns@peds.ufl.edu; Phone: 352-273-8259; Fax: 352-273-8342

Emmanuel Payen, PhD
CEA de Fontenay aux Roses, Institut des Maladies Emergentes et des Thérapies Innovantes, 18, route du Panorama - BP6, 92265 Fontenay aux Roses Cedex, FRANCE; E-mail: emmanuel.payen@cea.fr; Phone: + 33 146547055; Fax: +33 1 46547499

Philippe Leboulch, MD, PhD
CEA de Fontenay aux Roses, Institut des Maladies Emergentes et des Thérapies Innovantes, , 18 route du Panorama - BP6, 92265 Fontenay-aux-Roses, FRANCE; E-mail: pleboulch@rics.bwh.harvard.edu; Phone: +33 1 46548349; Fax: +33 1 46547499

Supplementary Figures

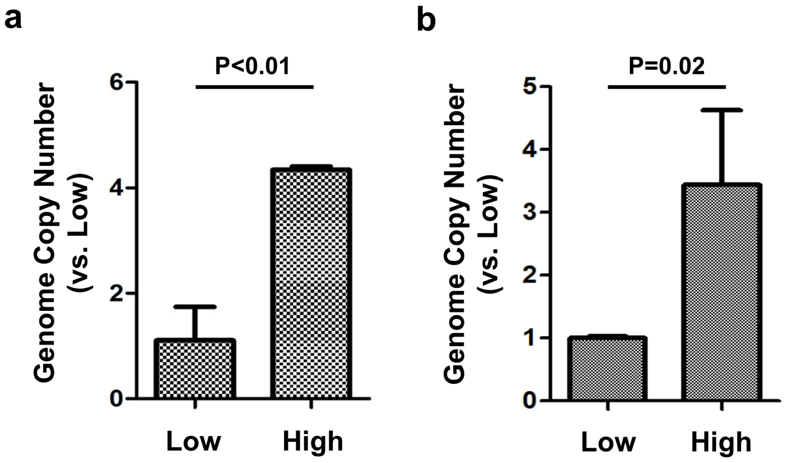

Supplementary Figure 1

Supplementary Figure 1. AAV vector genome copy numbers. Total DNAs were isolated from **(a)** K562 cells and **(b)** primary $CD34^+$ cells 2 hrs post-transduction with ssAAV6 vectors. The vector genome copy numbers/cell were determined by qPCR and were normalized to β-actin DNA copy number.

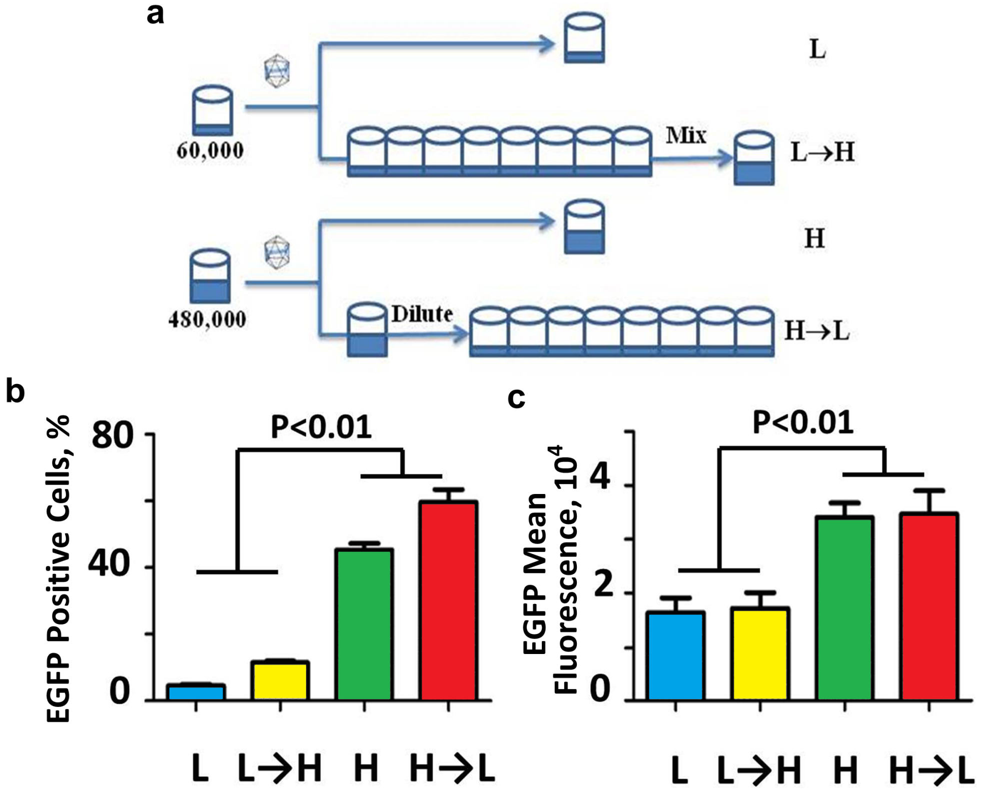

Supplementary Figure 2

Supplementary Figure 2. Effect of initial cell-cell contact. (a) Schematics of the experiment design. K562 cells (60,000 cells/50 μl) and 8 replicate cultures were transduced at low-density (L) with TM-scAAV6-CBAp-EGFP vectors at an MOI of 3,000 vgs/cell for 2 hrs and subsequently pooled together and centrifuged to reach high-density (L→H), and conversely, cells (480,000 cells/50 μl) were transduced at high-density (H), and 2 hrs post-transduction, were diluted to low-density (H→L). **(b)** Transgene expression in each cell population was analyzed by fluorescence microscopy 48 hrs post-transduction. **(c)** Mean fluorescence intensity of transgene expression.

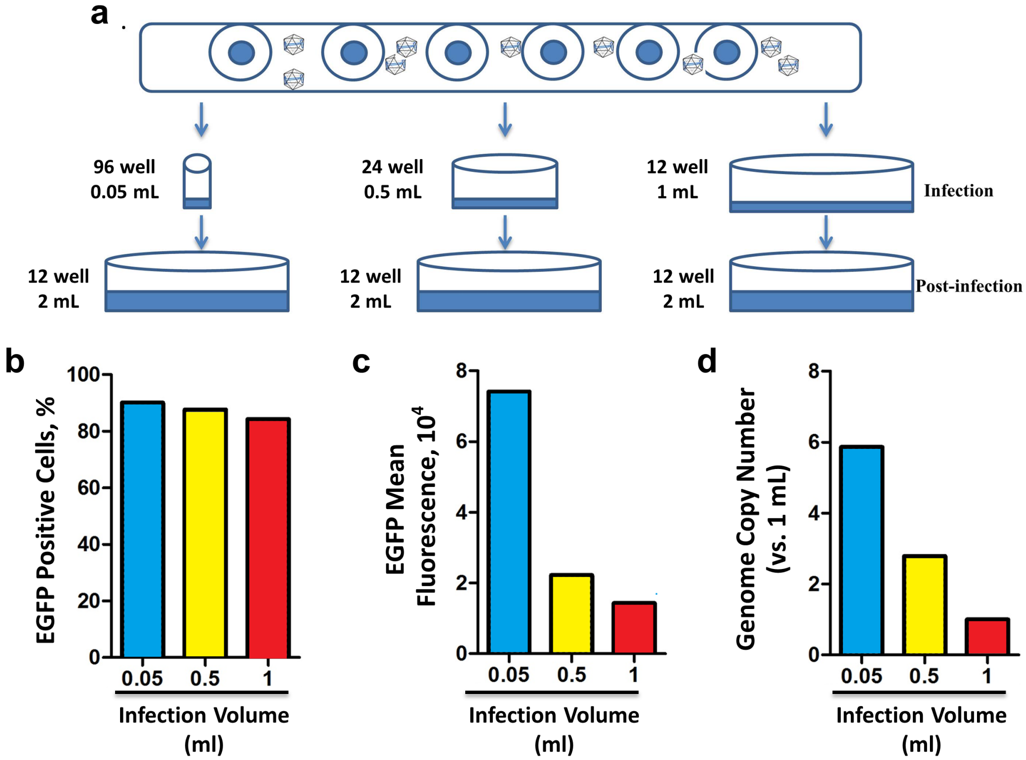

Supplementary Figure 3

Supplementary Figure 3. Effect of culture volume. (a) Schematics of the experiment design. A fixed number of K562 cells were transduced with viral vectors in various indicated volumes for 2 hrs and subsequently diluted to 2.0 ml. **(b)** Transgene expression in each cell population was analyzed by flow cytometry 48 hrs post-transduction. **(c)** Mean fluorescence intensity of transgene expression. **(d)** Vector genome copy numbers/cell were determined by qPCR and were normalized to β-actin DNA copy number as described above.

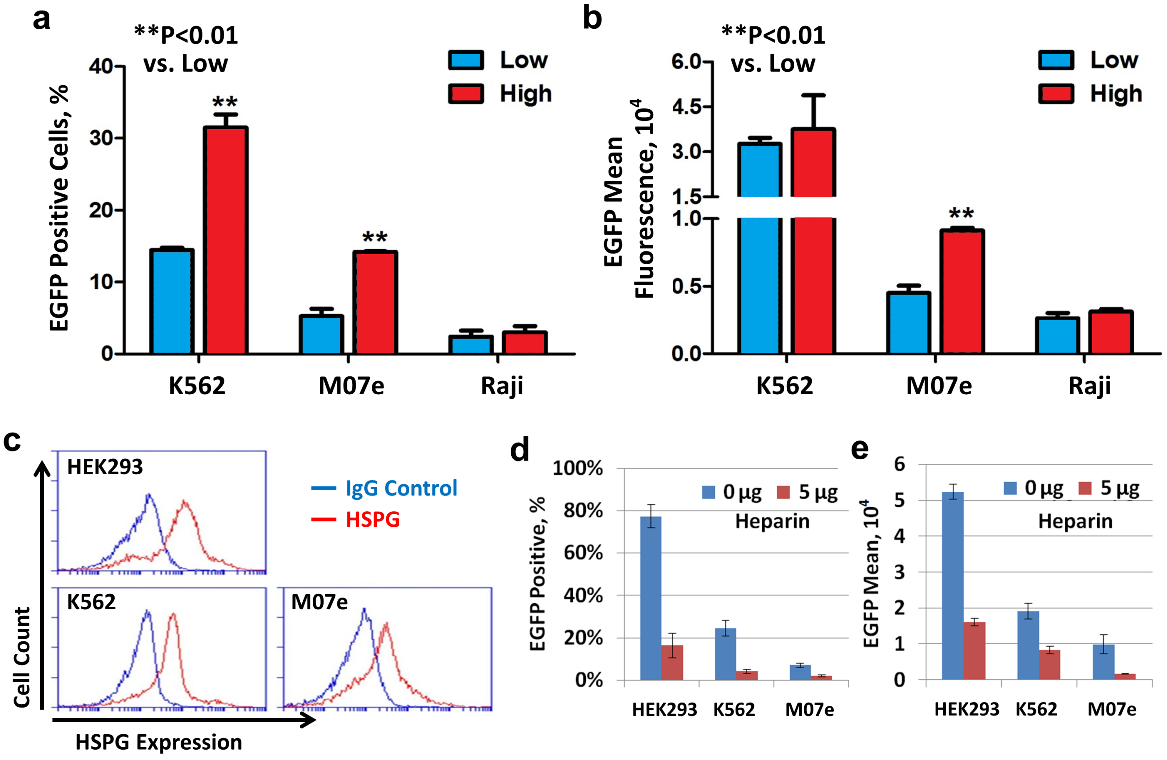

Supplementary Figure 4

Supplementary Figure 4. Transduction efficiency of AAV2 vectors in various human hematopoietic cell lines at low and high cell densities. (a) Human K562, M07e, and Raji cells were transduced with scAAV2-CBAp-EGFP vectors at an MOI of 5,000 vgs/cell at either low ($1x10^6$ cells/ml) or high ($1x10^7$ cells/ml) cell density. Transgene expression was analyzed by flow cytometry 48 hrs post-transduction. **(b)** Mean fluorescence intensity of transgene expression in each cell type. **(c)** FACS analyses of the level of expression of membrane heparan sulfate proteoglycan in various human cell types. **(d)** Transduction efficiency of AAV2 vectors in human cell lines in the absence or the presence of heparin. Each cell type was transduced with TM-scAAV6-CBAp-EGFP vector at an MOI of 5,000 vgs/cell at high ($1x10^7$ cells/ml) cell density, and transgene expression was analyzed by flow cytometry 48 hrs post-transduction. **(e)** Mean fluorescence intensity of transgene expression in each cell type

Supplementary Table 1: Transduction efficiency of WT and mutant AAV2 and AAV6 serotype vectors in primary human HSPCs from various donors.

	WT-AAV2	WT-AAV6	TM-AAV6-	WT-AAV2	QM-AAV2-	WT-AAV6	TM-AAV6-
	Low Cell Density			High Cell Density			
**Donor #1**	ND	1.3% 5,090	ND	ND	ND	19.4% 9,080	ND
**Donor #2**	1.4%, 4,184	9.8% 6,904	ND	7.2% 5,810	31.1% 15,672	27.0% 9,994	45.2% 25,373
**Donor #3**	ND	ND	28.6%, 8,246	ND	ND	ND	58.5% 23,846
**Donor #4**	4.4%, 3,720	9.5%, 6,512	ND	11.6%, 6,686	19.6%, 13,001	23.9%, 15,434	ND

Primary human bone marrow-derived $CD34^+$ cells from various donors were transduced in triplicate with the WT-AAV2, WT-AAV6, Y444+500+730F+T491T-AAV2 quadruple-mutant (QM), or Y705+731F+T492V-AAV6 triple-mutant (TM) scAAV-EGFP vectors at an MOI of 3,000 vgs/cell. Transgene expression was analyzed by flow cytometry 48 hrs post-transduction. Data are presented as % EGFP positive cells, and EGFP mean fluorescence intensities.

ND = Not done.
